# 2-Imino-3-(2-nitro­phen­yl)-1,3-thia­zolidin-4-one

**DOI:** 10.1107/S1600536811033812

**Published:** 2011-09-14

**Authors:** Muhammad Zia-ur-Rehman, Mark R. J. Elsegood, Muhammad Nadeem Arshad, Abdullah M. Asiri

**Affiliations:** a Applied Chemistry Research Centre, PCSIR Laboratories Complex, Lahore-54600, Pakistan; b Chemistry Department, Loughborough University, Loughborough LE11 3TU, England; c X-ray Diffraction and Crystallography Laboratory, Department of Physics, School of Physical Sciences, University of the Punjab, Quaid-e-Azam Campus, Lahore-54590, Pakistan; d The Center of Excellence for Advanced Materials Research, King Abdul Aziz University, Jeddah, PO Box 80203, Saudi Arabia.

## Abstract

In the title compound, C_9_H_7_N_3_O_3_S, the nitro and thia­zolidinone moieties are inclined with respect to the aromatic ring at dihedral angles of 9.57 (16) and 78.42 (4)°, respectively. In the crystal, N—H⋯O hydrogen bonding connects the mol­ecules along the *c* and *a* axes to form a two-dimensional polymeric network. A weak S⋯O inter­action [3.2443 (11) Å] and phenyl ring to phenyl ring off-set π⋯π stacking [with centroid–centroid separation of 3.6890 (7) Å and ring slippage of 1.479 Å] link the polymeric chains along the *b* and *a* axes, respectively.

## Related literature

For the biological activities of thia­zolidinones, see: Barreca *et al.* (2001 ▶); Shah & Desai (2007 ▶); Mehta *et al.* (2006 ▶); Vazzana *et al.* (2004 ▶); Wrobel *et al.* (2006 ▶). For related structures, see: Shahwar *et al.* (2009 ▶, 2011 ▶); Zhou *et al.* (2008 ▶). For graph-set notation, see: Bernstein *et al.* (1995 ▶). For the comparative C—C separation in graphite, see: Trucano & Chen (1975 ▶).
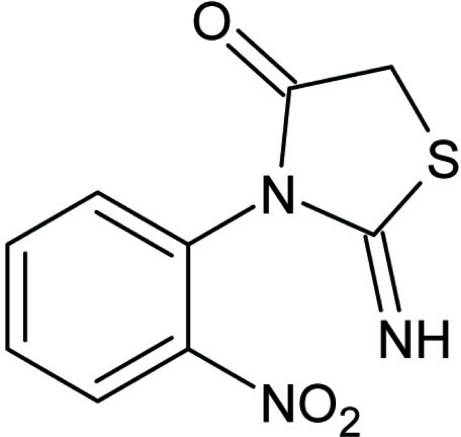


## Experimental

### 

#### Crystal data

C_9_H_7_N_3_O_3_S
*M* *_r_* = 237.24
Monoclinic, 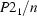

*a* = 7.3036 (5) Å
*b* = 16.4409 (10) Å
*c* = 8.2455 (5) Å
β = 102.1321 (9)°
*V* = 967.99 (11) Å^3^
*Z* = 4
Mo *K*α radiation
μ = 0.33 mm^−1^
*T* = 150 K
0.70 × 0.61 × 0.40 mm

#### Data collection

Bruker APEXII CCD diffractometer
Absorption correction: multi-scan (*SADABS*; Sheldrick, 2003 ▶) *T*_min_ = 0.802, *T*_max_ = 0.880
11000 measured reflections
2938 independent reflections
2675 reflections with *I* > 2σ(*I*)
*R*_int_ = 0.018

#### Refinement

*R*[*F* ^2^ > 2σ(*F* ^2^)] = 0.033
*wR*(*F* ^2^) = 0.093
*S* = 1.03
2938 reflections
148 parameters
H atoms treated by a mixture of independent and constrained refinement
Δρ_max_ = 0.47 e Å^−3^
Δρ_min_ = −0.25 e Å^−3^

### 

Data collection: *APEX2* (Bruker, 2007 ▶); cell refinement: *SAINT* (Bruker, 2007 ▶); data reduction: *SAINT*; program(s) used to solve structure: *SHELXS97* (Sheldrick, 2008 ▶); program(s) used to refine structure: *SHELXL97* (Sheldrick, 2008 ▶); molecular graphics: *SHELXTL* (Sheldrick, 2008 ▶); software used to prepare material for publication: *SHELXTL* and local programs.

## Supplementary Material

Crystal structure: contains datablock(s) I, global. DOI: 10.1107/S1600536811033812/ez2256sup1.cif

Structure factors: contains datablock(s) I. DOI: 10.1107/S1600536811033812/ez2256Isup2.hkl

Supplementary material file. DOI: 10.1107/S1600536811033812/ez2256Isup3.cml

Additional supplementary materials:  crystallographic information; 3D view; checkCIF report

## Figures and Tables

**Table 1:** Hydrogen-bond geometry (Å, °)

*D*—H⋯*A*	*D*—H	H⋯*A*	*D*⋯*A*	*D*—H⋯*A*
N1—H1⋯O1^i^	0.886 (18)	2.334 (18)	3.0337 (13)	135.9 (14)
N1—H1⋯O2^ii^	0.886 (18)	2.439 (17)	3.1416 (14)	136.5 (14)

Symmetry codes: (i) 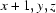; (ii) 
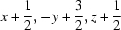
.

**Fig. 1.**
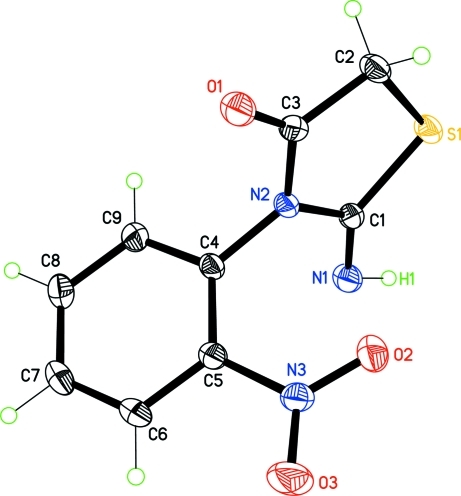
The labelled molecular structure of (I) showing 50% displacement ellipsoids.

**Fig. 2.**
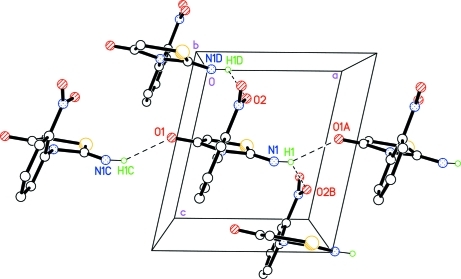
Packing plot parallel to b which shows the polymeric network through N—H···O hydrogen bonds (drawn as dashed lines).

**Fig. 3.**
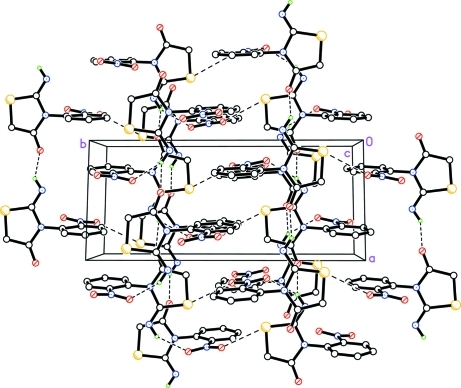
Packing plot viewed parallel to c showing N—H···O hydrogen bonds, weak S···O interactions (drawn as dashed lines) and stacked aromatic moieties.

**Fig. 4.**
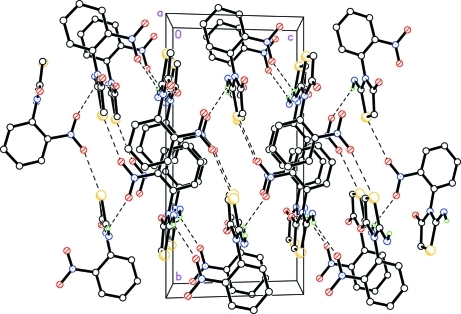
Packing plot viewed parallel to a showing N—H···O hydrogen bonds, weak S···O interactions (drawn as dashed lines) and stacked aromatic moieties

## supplementary crystallographic information

### Comment

Thiazolidinones are well known heterocyclic compounds, familiar for their anti-bacterial (Shah & Desai, 2007), anti-fungal (Mehta *et al.*, 2006), anti-HIV (Barreca *et al.*, 2001), anti-iflammatory (Vazzana *et al.*, 2004), anti-cancer (Zhou *et al.*, 2008) and FSH receptor agonist (Wrobel *et al.*, 2006) activities. Herein, we report the synthesis and crystal structure of a new example of this class of compound, I (Fig. 1 & Scheme).

The structure of the title compound correlates with the crystal structures of other thiazolidinones (Shahwar, *et al.*, 2009 & Shahwar, *et al.*, 2011). The nitro group is inclined at a dihedral angle of 9.57 (16)° with respect to the phenyl ring. The thiazolidinone ring is essentially planar with an r.m.s. deviation of 0.0144 Å with the maximum deviation at the carbon atoms (C2 = 0.0196 (6) Å & C3 = -0.0193 (7) Å). The dihedral angle between the two planes C4 to C9 and N1/C3/C2/S1/C1 is 78.42 (4)°. The amino group is involved in two unique N—H···O intermolecular hydrogen bonding interactions. The first results in zigzag C(8) chains (Bernstein *et al.*, 1995) along the *c* axis; the second in C(6) chains along the *a* axis (Table 1, Fig. 2). Weak S···O interactions link sheets together in the *b* direction with S1···O3 = 3.244 Å (Fig. 3). Off-set π···π stacking connects the chain along the *a* axis involving C5/C6/C7 with separations of *ca*. 3.41–3.53 Å (Figures 3 & 4). This is slightly longer than the *ca* 3.35 Å separation in graphite (Trucano & Chen, 1975).

### Experimental

A mixture of 1-isothiocyanato-2-nitrobenzene (8.0 mmoles), dichloromethane (20 ml), and anhydrous ammonium carbonate (8.0 mmoles) was stirred at room temperature under inert atmosphere (nitrogen) for 2 h. Paraformaldehyde (4 mmoles) was added to it portion wise, and the contents were allowed to stir for 10 h; cooled to 0°C, followed by addition of 0.5 *M* aqueous sodium hydroxide (20 ml) over 30 minutes. The cloudy solution was heated to reflux for 2 h and the reaction mixture was neutralized with dilute hydrochloric acid. The aqueous layer was extracted with ethyl acetate (3 *x* 30 ml); washed with water and brine and dried over sodium sulfate. Slow evaporation of the solvent furnished pale yellow crystals.

### Refinement

All the CH hydrogen atoms were located *via* a difference map and refined with a constrained, riding model with aromatic CH = 0.95 and CH_2_ 0.99 Å. The NH hydrogen has coordinates freely refined. *U*_iso_(H) was set to 1.2*U*_eq_ of that of the carrier atom..

### Crystal data

**Table:**

C_9_H_7_N_3_O_3_S	*F*(000) = 488
*M**_r_* = 237.24	*D*_x_ = 1.628 Mg m^−^^3^
Monoclinic, *P*2_1_/*n*	Mo *K*α radiation, λ = 0.71073 Å
Hall symbol: -P 2yn	Cell parameters from 6547 reflections
*a* = 7.3036 (5) Å	θ = 2.5–30.5°
*b* = 16.4409 (10) Å	µ = 0.33 mm^−^^1^
*c* = 8.2455 (5) Å	*T* = 150 K
β = 102.1321 (9)°	Block, light yellow
*V* = 967.99 (11) Å^3^	0.70 × 0.61 × 0.40 mm
*Z* = 4	

### Data collection

**Table:**

Bruker APEXII CCD diffractometer	2938 independent reflections
Radiation source: fine-focus sealed tube	2675 reflections with *I* > 2σ(*I*)
graphite	*R*_int_ = 0.018
ω rotation with narrow frames scans	θ_max_ = 30.5°, θ_min_ = 2.5°
Absorption correction: multi-scan (*SADABS*; Sheldrick, 2003)	*h* = −10→10
*T*_min_ = 0.802, *T*_max_ = 0.880	*k* = −23→23
11000 measured reflections	*l* = −11→11

### Refinement

**Table:**

Refinement on *F*^2^	Primary atom site location: structure-invariant direct methods
Least-squares matrix: full	Secondary atom site location: structure-invariant direct methods
*R*[*F*^2^ > 2σ(*F*^2^)] = 0.033	Hydrogen site location: inferred from neighbouring sites
*wR*(*F*^2^) = 0.093	H atoms treated by a mixture of independent and constrained refinement
*S* = 1.03	*w* = 1/[σ^2^(*F*_o_^2^) + (0.0513*P*)^2^ + 0.3974*P*] where *P* = (*F*_o_^2^ + 2*F*_c_^2^)/3
2938 reflections	(Δ/σ)_max_ = 0.001
148 parameters	Δρ_max_ = 0.47 e Å^−^^3^
0 restraints	Δρ_min_ = −0.25 e Å^−^^3^

### Special details

**Table:**

Geometry. All e.s.d.'s (except the e.s.d. in the dihedral angle between two l.s. planes) are estimated using the full covariance matrix. The cell e.s.d.'s are taken into account individually in the estimation of e.s.d.'s in distances, angles and torsion angles; correlations between e.s.d.'s in cell parameters are only used when they are defined by crystal symmetry. An approximate (isotropic) treatment of cell e.s.d.'s is used for estimating e.s.d.'s involving l.s. planes.
Refinement. Refinement of *F*^2^ against ALL reflections. The weighted *R*-factor *wR* and goodness of fit *S* are based on *F*^2^, conventional *R*-factors *R* are based on *F*, with *F* set to zero for negative *F*^2^. The threshold expression of *F*^2^ > σ(*F*^2^) is used only for calculating *R*-factors(gt) *etc*. and is not relevant to the choice of reflections for refinement. *R*-factors based on *F*^2^ are statistically about twice as large as those based on *F*, and *R*- factors based on ALL data will be even larger.

### Fractional atomic coordinates and isotropic or equivalent isotropic displacement parameters (Å^2^)

**Table:**

	*x*	*y*	*z*	*U*_iso_*/*U*_eq_	
C1	0.43457 (15)	0.75201 (6)	0.52262 (14)	0.0182 (2)	
N1	0.58325 (14)	0.79287 (6)	0.55992 (14)	0.0239 (2)	
H1	0.686 (2)	0.7635 (10)	0.564 (2)	0.029*	
S1	0.40353 (4)	0.646740 (16)	0.48418 (4)	0.02236 (9)	
C2	0.15109 (16)	0.65317 (6)	0.44907 (16)	0.0213 (2)	
H2A	0.0937	0.6319	0.3376	0.026*	
H2B	0.1044	0.6207	0.5330	0.026*	
C3	0.10139 (15)	0.74173 (6)	0.46228 (14)	0.0186 (2)	
O1	−0.05729 (12)	0.76769 (5)	0.43725 (12)	0.02667 (19)	
N2	0.25861 (12)	0.78966 (5)	0.50525 (12)	0.01726 (18)	
C4	0.24651 (14)	0.87451 (6)	0.53960 (13)	0.01660 (19)	
C5	0.26722 (14)	0.93712 (6)	0.42976 (13)	0.01709 (19)	
C6	0.25532 (15)	1.01822 (7)	0.47384 (14)	0.0206 (2)	
H6	0.2702	1.0601	0.3984	0.025*	
C7	0.22162 (16)	1.03764 (7)	0.62847 (15)	0.0233 (2)	
H7	0.2142	1.0930	0.6595	0.028*	
C8	0.19873 (17)	0.97645 (7)	0.73760 (15)	0.0243 (2)	
H8	0.1740	0.9899	0.8430	0.029*	
C9	0.21179 (16)	0.89517 (7)	0.69338 (14)	0.0215 (2)	
H9	0.1968	0.8535	0.7693	0.026*	
N3	0.30099 (14)	0.92086 (6)	0.26329 (12)	0.02117 (19)	
O2	0.33657 (15)	0.85158 (5)	0.22577 (12)	0.0294 (2)	
O3	0.29218 (19)	0.97834 (6)	0.16775 (13)	0.0414 (3)	

### Atomic displacement parameters (Å^2^)

**Table:**

	*U*^11^	*U*^22^	*U*^33^	*U*^12^	*U*^13^	*U*^23^
C1	0.0191 (5)	0.0140 (4)	0.0231 (5)	0.0032 (4)	0.0083 (4)	0.0021 (4)
N1	0.0180 (4)	0.0189 (4)	0.0361 (5)	0.0019 (3)	0.0085 (4)	0.0007 (4)
S1	0.02110 (15)	0.01369 (14)	0.03376 (17)	0.00281 (9)	0.00911 (11)	−0.00049 (10)
C2	0.0203 (5)	0.0130 (4)	0.0310 (6)	0.0007 (4)	0.0060 (4)	−0.0002 (4)
C3	0.0191 (5)	0.0148 (4)	0.0231 (5)	−0.0007 (4)	0.0072 (4)	0.0011 (4)
O1	0.0182 (4)	0.0202 (4)	0.0424 (5)	0.0015 (3)	0.0081 (3)	−0.0003 (4)
N2	0.0164 (4)	0.0119 (4)	0.0249 (4)	0.0013 (3)	0.0074 (3)	0.0004 (3)
C4	0.0153 (4)	0.0125 (4)	0.0224 (5)	0.0018 (3)	0.0049 (4)	0.0003 (4)
C5	0.0167 (4)	0.0150 (4)	0.0194 (4)	−0.0005 (3)	0.0035 (3)	0.0003 (4)
C6	0.0212 (5)	0.0138 (5)	0.0256 (5)	−0.0011 (4)	0.0020 (4)	0.0009 (4)
C7	0.0233 (5)	0.0157 (5)	0.0294 (6)	0.0019 (4)	0.0023 (4)	−0.0047 (4)
C8	0.0274 (5)	0.0219 (5)	0.0245 (5)	0.0043 (4)	0.0073 (4)	−0.0043 (4)
C9	0.0249 (5)	0.0181 (5)	0.0232 (5)	0.0032 (4)	0.0091 (4)	0.0018 (4)
N3	0.0243 (5)	0.0192 (4)	0.0201 (4)	−0.0040 (3)	0.0049 (3)	0.0003 (3)
O2	0.0430 (5)	0.0220 (4)	0.0257 (4)	0.0011 (4)	0.0125 (4)	−0.0036 (3)
O3	0.0751 (8)	0.0249 (5)	0.0263 (5)	−0.0035 (5)	0.0156 (5)	0.0074 (4)

### Geometric parameters (Å, °)

**Table:**

C1—N1	1.2589 (15)	C4—C5	1.4002 (14)
C1—N2	1.4064 (13)	C5—C6	1.3895 (14)
C1—S1	1.7655 (11)	C5—N3	1.4693 (14)
N1—H1	0.886 (18)	C6—C7	1.3857 (17)
S1—C2	1.8083 (12)	C6—H6	0.9500
C2—C3	1.5100 (14)	C7—C8	1.3831 (17)
C2—H2A	0.9900	C7—H7	0.9500
C2—H2B	0.9900	C8—C9	1.3938 (15)
C3—O1	1.2113 (14)	C8—H8	0.9500
C3—N2	1.3762 (14)	C9—H9	0.9500
N2—C4	1.4299 (13)	N3—O2	1.2225 (13)
C4—C9	1.3866 (15)	N3—O3	1.2234 (13)
			
N1—C1—N2	120.86 (10)	C5—C4—N2	124.72 (9)
N1—C1—S1	129.69 (9)	C6—C5—C4	121.00 (10)
N2—C1—S1	109.45 (8)	C6—C5—N3	116.81 (9)
C1—N1—H1	113.5 (11)	C4—C5—N3	122.19 (9)
C1—S1—C2	93.42 (5)	C7—C6—C5	119.65 (10)
C3—C2—S1	107.29 (7)	C7—C6—H6	120.2
C3—C2—H2A	110.3	C5—C6—H6	120.2
S1—C2—H2A	110.3	C8—C7—C6	120.02 (10)
C3—C2—H2B	110.3	C8—C7—H7	120.0
S1—C2—H2B	110.3	C6—C7—H7	120.0
H2A—C2—H2B	108.5	C7—C8—C9	120.19 (11)
O1—C3—N2	123.96 (10)	C7—C8—H8	119.9
O1—C3—C2	124.29 (10)	C9—C8—H8	119.9
N2—C3—C2	111.75 (9)	C4—C9—C8	120.65 (11)
C3—N2—C1	117.99 (9)	C4—C9—H9	119.7
C3—N2—C4	121.79 (9)	C8—C9—H9	119.7
C1—N2—C4	120.16 (9)	O2—N3—O3	122.83 (11)
C9—C4—C5	118.48 (10)	O2—N3—C5	119.50 (9)
C9—C4—N2	116.80 (9)	O3—N3—C5	117.68 (10)
			
N1—C1—S1—C2	178.92 (12)	C1—N2—C4—C5	80.50 (14)
N2—C1—S1—C2	−1.26 (9)	C9—C4—C5—C6	0.66 (16)
C1—S1—C2—C3	2.58 (9)	N2—C4—C5—C6	−179.31 (10)
S1—C2—C3—O1	176.24 (10)	C9—C4—C5—N3	−178.92 (10)
S1—C2—C3—N2	−3.36 (12)	N2—C4—C5—N3	1.11 (16)
O1—C3—N2—C1	−176.90 (11)	C4—C5—C6—C7	−0.29 (16)
C2—C3—N2—C1	2.70 (14)	N3—C5—C6—C7	179.30 (10)
O1—C3—N2—C4	6.11 (17)	C5—C6—C7—C8	−0.45 (17)
C2—C3—N2—C4	−174.29 (9)	C6—C7—C8—C9	0.81 (18)
N1—C1—N2—C3	179.21 (11)	C5—C4—C9—C8	−0.29 (17)
S1—C1—N2—C3	−0.64 (12)	N2—C4—C9—C8	179.68 (10)
N1—C1—N2—C4	−3.75 (16)	C7—C8—C9—C4	−0.44 (18)
S1—C1—N2—C4	176.40 (8)	C6—C5—N3—O2	170.66 (11)
C3—N2—C4—C9	77.45 (13)	C4—C5—N3—O2	−9.75 (16)
C1—N2—C4—C9	−99.47 (12)	C6—C5—N3—O3	−9.25 (16)
C3—N2—C4—C5	−102.57 (13)	C4—C5—N3—O3	170.35 (11)

### Hydrogen-bond geometry (Å, °)

**Table:**

*D*—H···*A*	*D*—H	H···*A*	*D*···*A*	*D*—H···*A*
N1—H1···O1^i^	0.886 (18)	2.334 (18)	3.0337 (13)	135.9 (14)
N1—H1···O2^ii^	0.886 (18)	2.439 (17)	3.1416 (14)	136.5 (14)

Symmetry codes: (i) *x*+1, *y*, *z*; (ii) *x*+1/2, −*y*+3/2, *z*+1/2.

## References

[bb1] Barreca, M. L., Chimirri, A., Luca, L. D., Monforte, A. M., Monforte, P., Rao, A., Zappalà, M., Balzarini, J., Clercq, E. D., Pannecouque, C. & Witvrouw, M. (2001). *Bioorg. Med. Chem. Lett.* **11**, 1793–1796. 10.1016/s0960-894x(01)00304-3 11425562

[bb2] Bernstein, J., Davis, R. E., Shimoni, L. & Chang, N.-L. (1995). *Angew. Chem. Int. Ed. Engl.* **34**, 1555–1573.

[bb3] Bruker (2007). *APEX2* and *SAINT* Bruker AXS Inc., Madison, Wisconsin, USA.

[bb4] Mehta, P. D., Sengar, N. P., Subrahmanyam, E. V. S. & Satyanarayana, D. (2006). *Indian J. Pharm. Sci.* **68**, 103–106.

[bb5] Shah, T. J. & Desai, V. A. (2007). *Arkivoc*, **xiv**, 218–228.

[bb6] Shahwar, D., Tahir, M. N., Raza, M. A., Ahmad, N. & Aslam, S. (2011). *Acta Cryst.* E**67**, o133. 10.1107/S1600536810051548 PMC3050282 21522643

[bb7] Shahwar, D., Tahir, M. N., Raza, M. A. & Iqbal, B. (2009). *Acta Cryst.* E**65**, o2917. 10.1107/S1600536809044304 PMC2971373 21578495

[bb8] Sheldrick, G. M. (2003). *SADABS* University of Göttingen, Germany.

[bb9] Sheldrick, G. M. (2008). *Acta Cryst.* A**64**, 112–122. 10.1107/S0108767307043930 18156677

[bb10] Trucano, P. & Chen, R. (1975). *Nature* *(London)*, **258**, 136–137.

[bb11] Vazzana, I., Terranova, E., Mattioli, F. & Sparatore, F. (2004). *Arkivoc*, **v**, 364–374.

[bb12] Wrobel, J., Jetter, J., Kao, W., Rogers, J., Di, L., Chi, J., Peréz, M. C., Chen, G.-C. & Shen, E. S. (2006). *Bioorg. Med. Chem.* **14**, 5729–5741. 10.1016/j.bmc.2006.04.012 16675221

[bb13] Zhou, H., Wu, S., Zhai, S., Liu, A., Sun, Y., Li, R., Zhang, Y., Ekins, S., Swaan, P. W., Fang, B., Zhang, B. & Yan, B. (2008). *J. Med. Chem.* **51**, 1242–125. 10.1021/jm7012024 18257542

